# Delayed Presentation of Malignancy-Associated Pseudoachalasia of the Gastric Cardia

**DOI:** 10.7759/cureus.54040

**Published:** 2024-02-11

**Authors:** Clive J Miranda, Farhan Azad, Ross R Moyer, Sasikanth N Ravi, Gina M Sparacino

**Affiliations:** 1 Internal Medicine, University at Buffalo, Buffalo, USA; 2 Pulmonology, Saint Peter’s University Hospital, New Brunswick, USA; 3 Internal Medicine, Abrazo Community Health Network, Phoenix, USA; 4 Gastroenterology and Hepatology, University at Buffalo, Buffalo, USA

**Keywords:** endoscopy, gastroesophageal junction (gej), esophageal motility disorder, achalasia, gastric adenocarcinoma, gastric cardia, endoscopic ultrasound (eus), gastric malignancy, pseudoachalasia

## Abstract

Pseudoachalasia is a condition in which symptoms, manometry, and imaging findings highly resemble primary achalasia but has a secondary etiology. The majority of patients with pseudoachalasia have the condition as the result of a malignancy, most often at the gastroesophageal junction. There may be issues with timely identification of this malignancy as symptoms are often obscure with diagnostic testing yielding nonspecific results. We describe a case of a 65-year-old diabetic female smoker with a four-month history of intractable vomiting, abdominal pain, and weight loss who was belatedly found to have an adenocarcinoma at the gastric cardia necessitating a total gastrectomy and chemotherapy administration. The case educates clinicians on the clinical alarm symptoms related to malignant pseudoachalasia and stresses the paramount importance of performing a timely esophagogastroduodenoscopy in all cases of achalasia, even with seemingly normal imaging, to rule out pseudoachalasia related to malignancy.

## Introduction

Pseudoachalasia is an esophageal motility disorder for which clinical and diagnostic features resemble idiopathic achalasia. First suggested by Howarth in 1919, patients with pseudoachalasia have distal esophageal narrowing with proximal dilation due to causes other than achalasia-related denervation of the myenteric ganglion cells, resulting in retrosternal pain, dysphagia, emesis, and weight loss developing over a short timeframe [1].

The incidence and prevalence of pseudoachalasia are not well known due to the lack of large case series on this pathology. However, it is estimated that approximately 2-4% of patients with manometric findings of achalasia suffer from pseudoachalasia [2,3]. In roughly 70% of patients with pseudoachalasia, the etiology is a primary or secondary malignancy, with primary malignancies comprising 54-70% of cases and secondary malignancies comprising 6% [4-8]. Other causes of pseudoachalasia comprise around 12% of cases and include lymphoproliferative disorders, amyloidosis, sarcoidosis, paraneoplastic diseases, pancreatic pseudocysts, Chagas disease, thoracic aortic aneurysm, and secondary to surgical procedures at the gastroesophageal junction (GEJ), although the list of etiologies is far from exhaustive [9].

The most common primary malignancy is a carcinoma of the esophagus or the cardia with stricturing at the GEJ due to mass effect. Other mechanisms of tumor involvement include: (a) tumor cells infiltrating the inhibitory neurons of the esophageal myenteric plexus and rendering them nonfunctional, (b) neuronal degeneration distant from the primary tumor site with reduction in ganglion cells in the dorsal nucleus of the vagus nerve or the in the vagus nerve itself, and (c) paraneoplastic patterns involving the interaction of tumor factors with the neuronal plexus of the esophagus without direct tumor invasion itself [4-6,10-13]. With clinical, radiographic, and manometric findings often unable to differentiate achalasia from pseudoachalasia, endoscopy is paramount in definitively ruling out pseudoachalasia in such cases.

Our case describes a 65-year-old female with prolonged symptoms of abdominal pain, emesis, and unintentional weight loss with minimal diagnostic workup at outside facilities, who was found to have a malignant tumor of the gastric cardia.

## Case presentation

A 65-year-old female with type II diabetes mellitus, hypertension, and tobacco use disorder presented to our county hospital's emergency department (ED) with complaints of persistent abdominal pain, intractable nausea and vomiting, and intolerance to oral intake that had been progressing for the past four months with an accompanying unintentional 40 lb weight loss. Prior to the preceding four months, she denied similar symptoms.

She had several presentations to the ED over the preceding months with limited workup undertaken which was unrevealing. One month into her symptoms, she had her first ED visit for which she was assumed to have gastroesophageal reflux disease and prescribed pantoprazole. This failed to resolve her symptoms and she visited another ED again after a further month. She was prescribed antibiotics this time with the presumption this was a *Helicobacter pylori* infection. A barium esophagram at yet another ED one month prior to her presentation with us was normal. Five days prior to the current presentation, she underwent a laparoscopic cholecystectomy for symptomatic cholelithiasis at a rural hospital. Of note, the patient only had hypertension as a comorbidity, was a retired secretary, had no family history of malignancy, and had no impairing socioeconomic or psychosocial history. There was no genetic analysis performed and she had a normal BMI, did not abuse alcohol, and had a healthy well-balanced diet with minimal cholesterol, salt-preserved foods, and carbonated/soft drinks.

**Figure 1:**
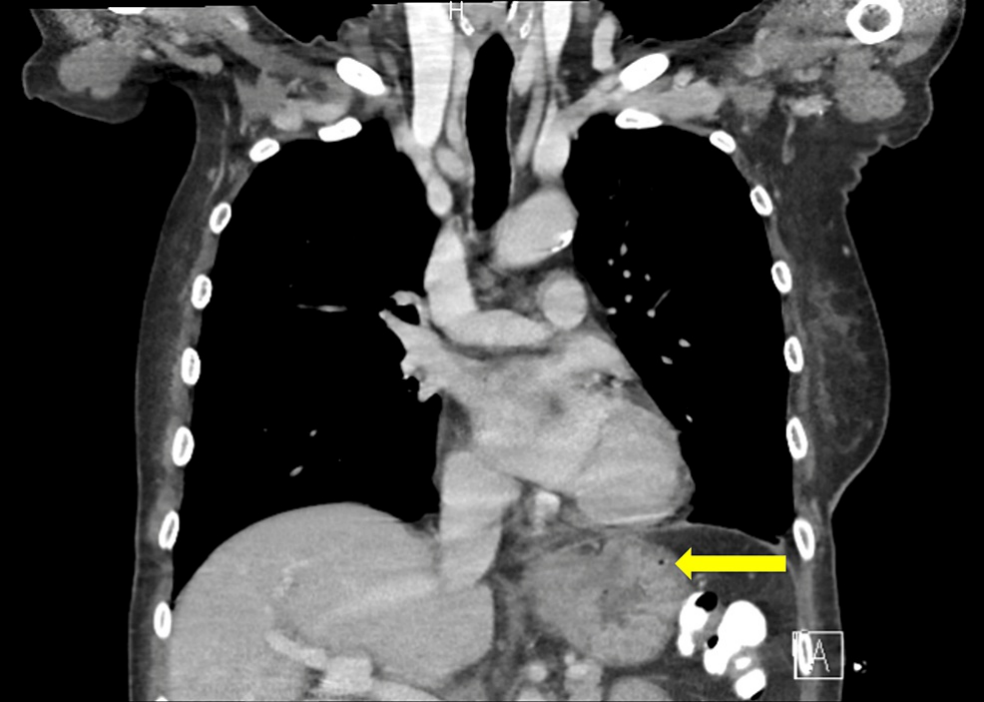
Coronal view showing mass-like mural thickening at the gastroesophageal junction (arrow) with diffuse distension of the thoracic esophagus raising the possibility of an obstruction.

**Figure 2:**
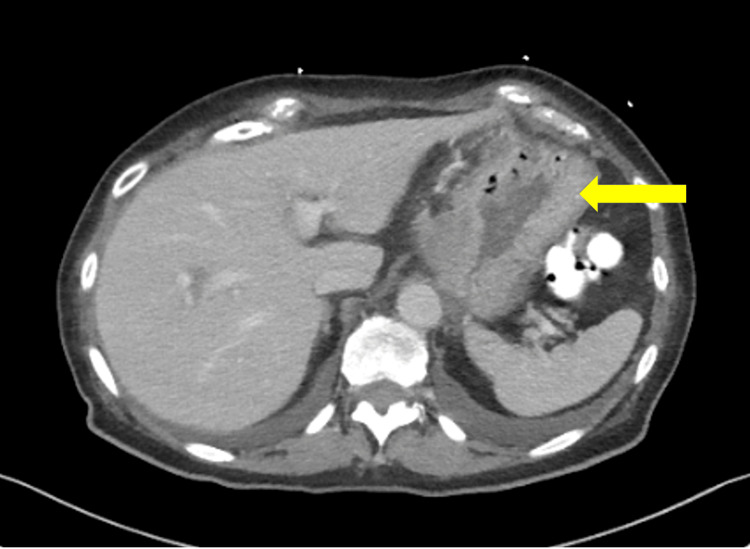
Transverse view showing focal gastroesophageal junction junction mural thickening, suspicious for a mass.

Workups for Epstein-Barr virus and *H. pylori* performed at other rural hospitals were negative. Persistent nausea and post-op vomiting prompted another rural ED visit one day prior to gastroenterology consult being placed at that institution. Electrolytes were corrected at the time and she was discharged home. The patient presented the following day again for persistent vomiting and inability to tolerate oral intake. She was hemodynamically stable; however, she was found to have hypernatremia with a sodium level of 160mmol/L and hypokalemia with a potassium level of 2.9mmol/L. Computed tomography (CT) revealed circumferential thickening of the distal esophagus (Figures 1, 2), prompting placement of gastroenterology consultation, and the patient subsequently underwent esophagogastroduodenoscopy (EGD), revealing fluid with some solid components in the distal esophagus. EGD was aborted due to the high risk of aspiration given these findings.

**Figure 3:**
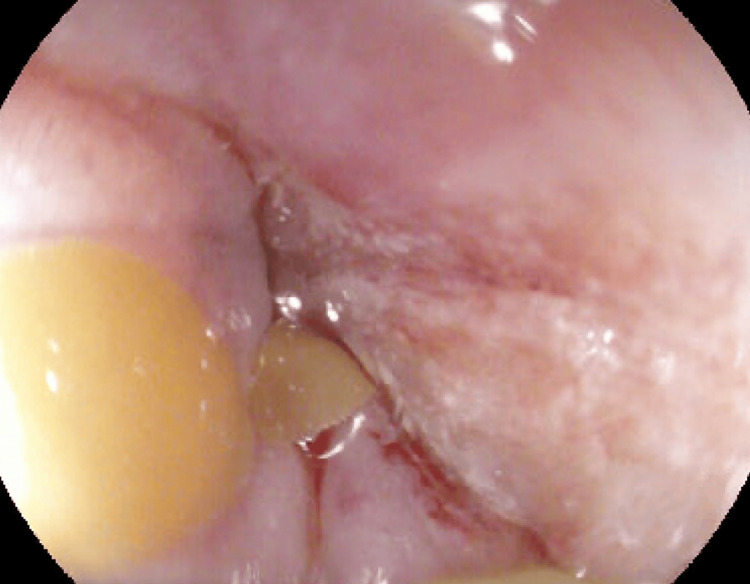
Pre-dilation showing distal esophageal stricture

**Figure 4:**
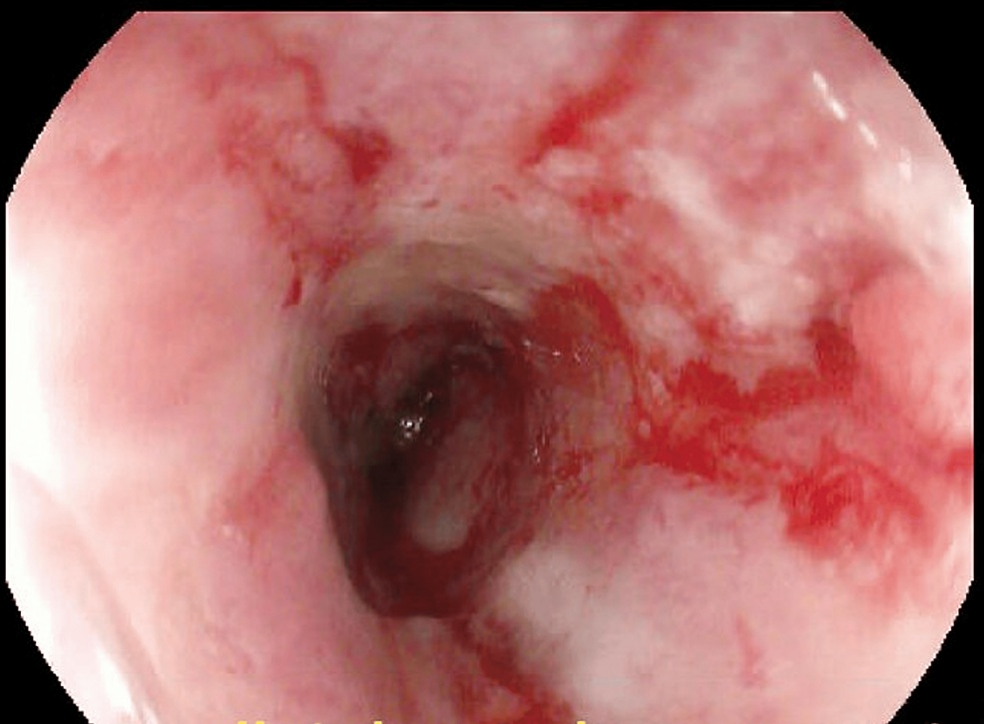
Esophagus post-dilation to the maximum of 12mm

**Figure 5:**
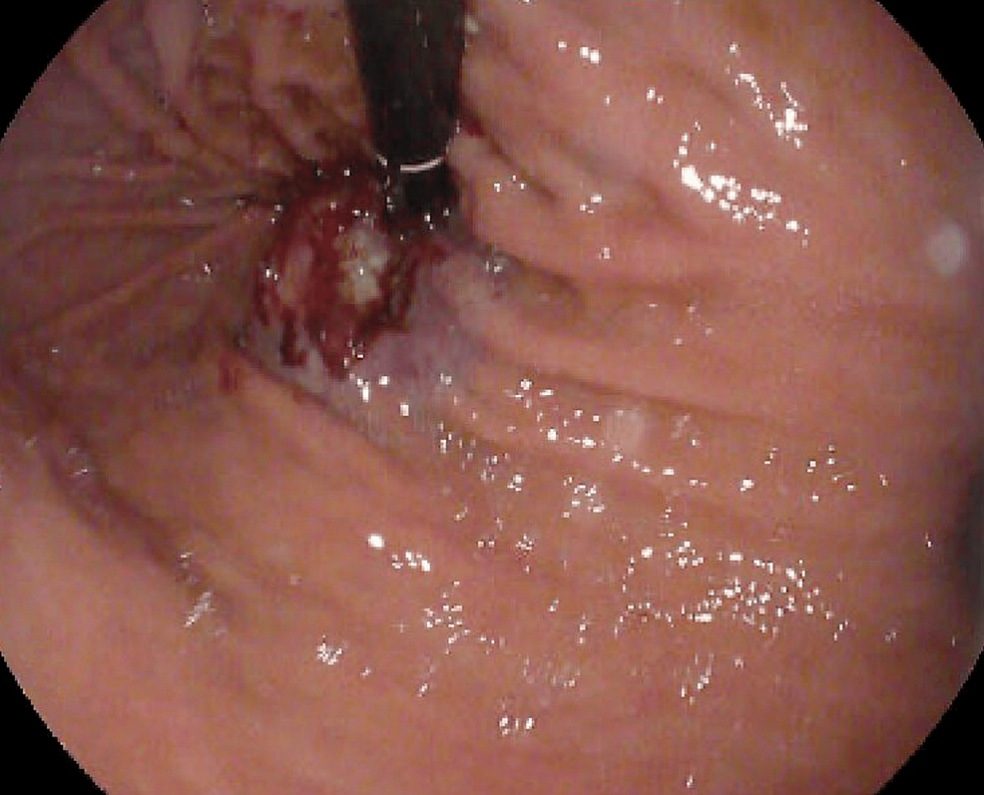
Retroflexion revealing a large ulcerated malignant mass

**Figure 6:**
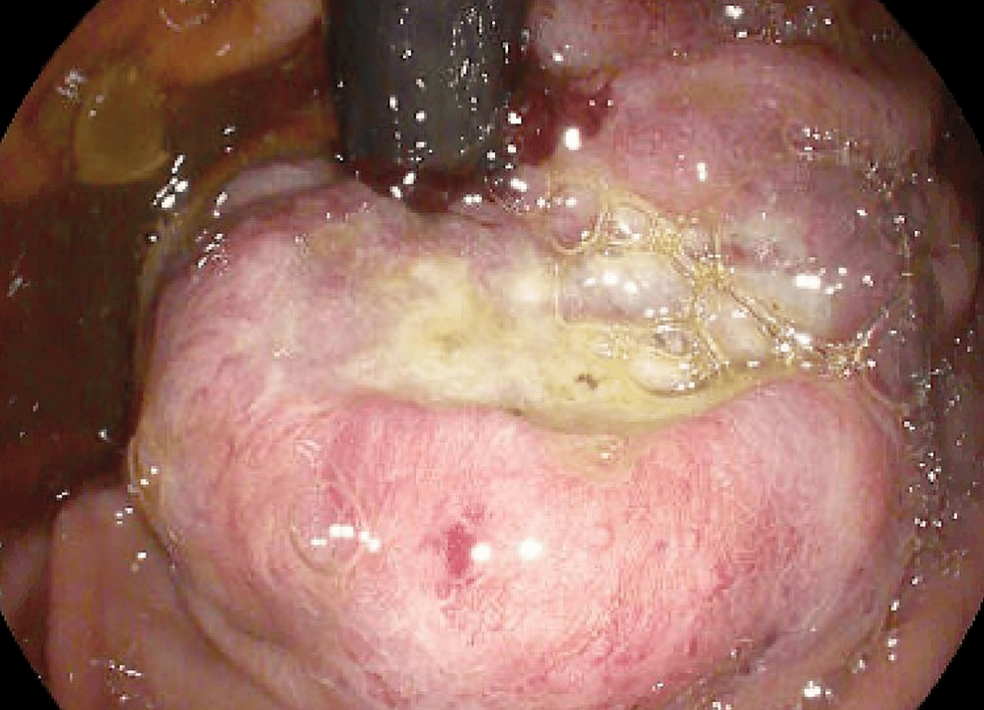
Closer inspection of large ulcerated malignant mass upon retroflexion

The following day, repeat EGD was undertaken in the operating room with intubation of the patient for airway protection prior to the procedure. The repeat EGD again revealed liquid with some solid components in the distal esophagus which was aggressively suctioned. There was difficulty advancing the endoscope through the distal esophagus due to narrowing and thus serial dilation was performed to a maximum dilation of 12 mm with successful traversing of the endoscope through the distal esophagus thereafter (Figures 3, 4). The endoscope was then advanced into the stomach, which was normal until retroflexion of the scope was performed, revealing a large, ulcerated mass at the gastric cardia, measuring 3cm in greatest diameter (Figures 5, 6).

**Figure 7:**
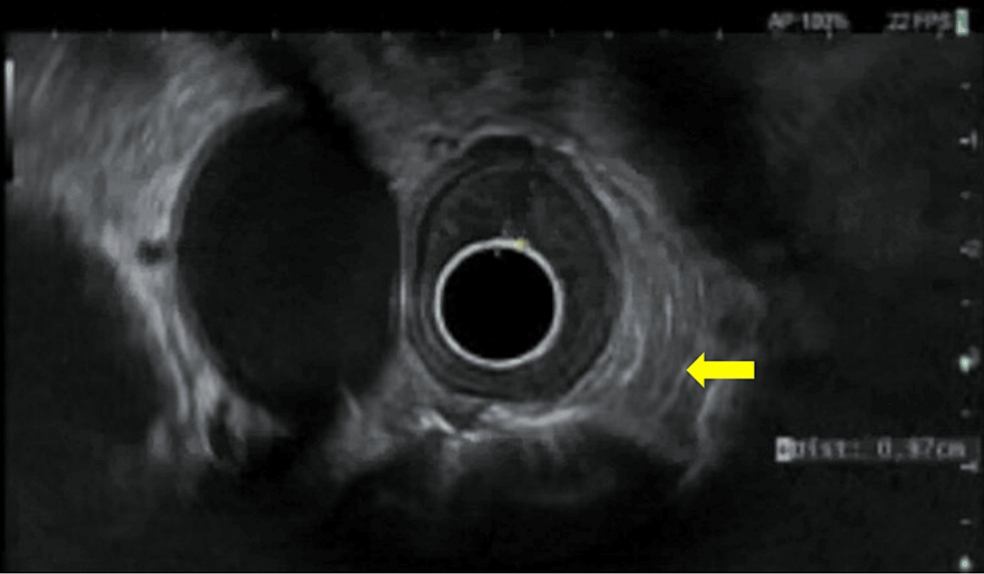
Endoscopic ultrasound revealing gastroesophageal junction mass (arrow) with invasion into muscularis propria, consistent with known adenocarcinoma from previous pathology, stage T3N1Mx

Biopsies were obtained using cold forceps and sent to pathology for histology which revealed moderately differentiated adenocarcinoma. The patient then underwent EGD with endoscopic ultrasound (EUS) seven days later for staging and possible esophageal stenting. EGD revealed severe, malignant-appearing, intrinsic stenosis measuring 2mm (inner diameter) x 3cm (length) that required scope downsizing and subsequent dilation performed with through-the-scope (TTS) balloon dilator up to 12mm. Food residue in the distal esophagus was removed using a Roth net and a large, ulcerated, non-circumferential mass without bleeding or stigmata of recent bleeding was observed in the gastric cardia with esophageal extension, classified as Siewert III. No periprocedural stenting was performed. EUS revealed a known irregular mass at the GEJ consistent with adenocarcinoma, measuring up to 14cm in thickness, with invasion into the muscularis propria (Figure 7). A few malignant-appearing lymph nodes in the lower paraesophageal mediastinum and peritumoral level were seen. The mass was staged as T3N1Mx by endosonographic criteria.

Two weeks later, she underwent a feeding jejunostomy tube placement as well as a mediport placement and was started on four cycles of neoadjuvant chemotherapy thereafter. Peritoneal washings were negative for malignancy. Follow-up positron emission tomography (PET) scan showed no definite nodal or distant metastases outside of her hypermetabolic gastric mass. Four months later, she underwent a laparoscopic total gastrectomy with adjuvant chemotherapy for three months. Left and right peritoneal washings were sent to pathology in approximately 30mL of cloudy pink fluid. For the left peritoneal washing, one liquid-based thin-layer cytologic slide was prepared for Papanicolaou staining and microscopic examination. For the right peritoneal washing, one Papanicolaou stained liquid-based thin-layer cytologic slide and one H&E-stained cell block slide were prepared for microscopic examination. These were both negative. However, immunostaining showed positivity for CK7, CK20, and CDX2 in tumor cells. Human epidermal growth factor receptor 2 (HER2) immunostaining was 2+ but HER2 by fluorescence in situ hybridization (FISH) was negative (HER2/CEN 17 ratio = 0.9). Resection margins were negative and there was no necrosis or lymphovascular/perineural invasion. The patient felt well since completing her chemotherapy regimen. Initially intolerant to oral intake, she had begun to gain more of an appetite towards the end of her adjuvant chemotherapy and has been physically active without fatigue, tolerating solid foods without issue. Her jejunostomy tube was removed four months after gastrectomy and she returned to working life with activity as tolerated. She was advised to follow up in the clinic as needed with her mediport to be removed one year after initial placement.

## Discussion

Pseudoachalasia is an uncommon, underdiagnosed disease with remarkable similarities to achalasia, thereby making an accurate diagnosis very challenging. Howarth first suggested the phenomenon in 1919 as a dilatation of the esophagus without anatomical stenosis [1]. This was then further expanded upon by Ogilvie in 1947 as a submucosal infiltration of the lower esophageal sphincter and cardia by a carcinoma [13]. Pseudoachalasia accounts for approximately 5% of patients with suspected achalasia, and primary and secondary malignancies account for the majority of pseudoachalasias [4,5]. It appears that the main cause of pseudoachalasia is an adenocarcinoma at the GEJ; however, similar effects have been also reported with multiple other cancers such as lung, pancreatic, breast, and cervical cancers, pleural mesothelioma, and multiple myeloma [14-21]. Nonmalignant causes of pseudoachalasia include, but are not limited to, amyloidosis, sarcoidosis, thoracic aortic aneurysm, systemic mastocytosis, Chagas disease, neurofibromatosis, histiocytosis-x, and Fabry disease with the accumulation of lysosomal Gb3 [6,22-29]. Anti-reflux surgery can also lead to pseudoachalasia due to misdiagnosed idiopathic achalasia with dysphagia symptoms postoperatively, underlying gastroesophageal reflux leading to achalasia, and an excessively tight fundic wrap or scar tissue development postoperatively. Schizas, et al. in 2020 compiled the first pseudoachalasia systematic review showing the range of malignant and benign pathologies that can lead to the dysmotility phenomenon [11].

Three main mechanisms of pseudoachalasia have been reported in the literature. The most common type is a malignant stricture near or at the GEJ that acts as a mechanical barrier to food. A less common type is a direct invasion of the inhibitory neurons of the lower esophageal sphincter invasion by tumor cells. Paraneoplastic processes are the third mechanism and encompass an interaction of tumor factors with the esophageal myenteric plexus. The main case series and case reports of malignant pseudoachalasia were summarized by Tustumi et al. in 2021 [12]. It was easily identified on upper endoscopy in our patient that the extraluminal gastric adenocarcinoma encroaching on the esophageal lumen was the pathogenic process by which her pseudoachalasia manifested.

It is important to recognize the delay in diagnosing our patient’s malignancy and the unremarkable diagnostic workup initially done at the start of her four-month period of symptoms. The nausea and abdominal pain could initially have been attributed to gastroesophageal reflux, especially considering the patient’s history of smoking, although this was unrelieved by proton pump inhibitors. Three months into her symptoms, a barium esophagram was still negative. It is likely the two-dimensional nature of barium esophagram imaging obscured the mass from view from the angle the diagnostic test was taken, thereby hindering a more comprehensive workup for malignancy. Nonetheless, it appears that the workup performed at outside hospitals relied heavily on non-invasive diagnostic modalities and leaned towards avoiding endoscopy as much as possible, with the assumption that her underlying diagnosis was hitherto as unremarkable as her workup.

Early identification of malignant pseudoachalasia is paramount to avoid delayed or inappropriate treatment. However, differentiating between achalasia and pseudoachalasia is highly challenging due to the high nature of comparability between clinical features and diagnostic modalities of the two conditions. Multiple studies in the past suggested the following clinical features in differentiating between the two conditions: age ≥55 years, duration of symptoms ≤6 months (some advocate for ≤12 months), and significant weight loss at presentation ≥10kg [3,4,6,10,30-32]. These criteria certainly applied to our patient. However, despite these criteria making pseudoachalasia more likely, subsequent analysis showed they have a poor predictive value overall and no greater than 18% [32]. In 2017, however, Ponds et al. used the Eckardt symptom score and demonstrated a significant and more discrete separation between malignant pseudoachalasia patients and those from other causes [33].

While clinical risk factors are useful in determining the presence of malignant pseudoachalasia, it is important to consider them alongside diagnostic risk factors as well. At our institution, the patient first underwent a CT scan showing circumferential thickening of the distal esophagus. CT imaging has been shown to be a useful diagnostic modality to discriminate achalasia and pseudoachalasia, often revealing an asymmetric esophageal wall thickening, a mass around the GEJ, and mediastinal lymphadenopathy in the case of the latter [34]. Metastases can also be identified as well as tumors causing a paraneoplastic achalasia-like process. Some studies, however, note that the capability of CT to detect malignancy is in only 42% of patients with malignant pseudoachalasia [30]. It is important to note, however, that a dilated distal esophagus is located in a transverse plane, and, as is often the case in patients with advanced-stage idiopathic achalasia, a CT scan does not represent a true “cross-section” and might falsely suggest a “pseudotumor” [35].

Conventional diagnostic imaging for achalasia such as manometry and barium esophagram are of negligible value in differentiating between achalasia and pseudoachalasia. In both conditions, there is absence of peristalsis and a normal to high basal pressure of the lower esophageal sphincter on manometry [5,30,36]. Nonetheless, there are reports that the presence of peristalsis in some swallows increases the suspicion of pseudoachalasia, though short-segment achalasia also has descriptions of peristalsis [37]. A barium esophagram could in theory show the “bird’s beak appearance” suspicious for a malignant lesion. However, the width of the esophagus or cardia often appears similar in both idiopathic achalasia and pseudoachalasia and the classic “bird’s beak appearance” could also be present in both conditions. Subsequent studies employed specific diagnostic criteria on esophagram, showing that a narrowed distal esophageal segment of ≥3.5cm with asymmetry and filling defects or an esophageal diameter of ≤4cm at its widest point was suggestive of pseudoachalasia and should prompt the search for possible malignancy given the clinical context [6,38,39]. However, none of this pertained to our patient who had a grossly normal esophagram three months into her symptoms.

Endoscopy remains likely the most sensitive diagnostic modality to uncover pseudoachalasia. In particular, difficulty in passing the endoscope into the stomach should prompt one to be suspicious of an obstructive malignancy at the GEJ or gastric cardia [2,30]. Biopsies should always be taken if traversing the GEJ proves challenging. In the case of our patient, moderately differentiated adenocarcinoma proved to be the etiology of her mechanical obstruction. However, even endoscopy has its limitations in diagnosing malignant pseudoachalasia. Tumors of the cardia or esophagus can be detected via endoscopy only if the malignancy crosses or disrupts the mucosal layer [30]. In a study by Kahrilas et al. in 1987, only 66% of patients with malignant pseudoachalasia were diagnosed with malignancy correctly by endoscopy and biopsy [5]. In another study by Lübke et al. in 1988, only 10% of all biopsies obtained in pseudoachalasia patients suggested a malignant presence [40]. In the largest cohort of malignant pseudoachalasia patients by Ponds et al. in 2017, not one patient had their malignancy revealed on initial endoscopy [30]. Often, a second or third endoscopy is necessary to diagnose a malignancy.

EUS is currently the most specific diagnostic modality available to identify suspected malignant pseudoachalasia. The American College of Gastroenterology advises performing EUS to differentiate between idiopathic achalasia and that associated with malignancy [41]. EUS can both rule out an infiltrating tumor (in the case of malignant pseudoachalasia) and also provide supportive evidence of idiopathic achalasia when a thickened circular muscle layer is seen [41,42]. Biopsies can be taken, there is no risk of exposure to radiation, and the procedure can be performed in conjunction with endoscopy or pneumodilation [42,43]. EUS on our patient showed the suspected adenocarcinoma with staging T3N1Mx. It is highly likely that a lower-stage cancer would have been identified had a more timely and comprehensive workup been initiated earlier on in the patient’s course of symptoms, highlighting the importance of addressing the clinical risk factors of malignant pseudoachalasia promptly.

## Conclusions

The diagnosis of malignant pseudoachalasia remains a challenge even for the most experienced physicians. Had more attention been given to our patient’s non-alleviating symptoms in the outside workups, her cancer diagnosis may not have been so extensive, and less aggressive surgical and oncological treatments may have been appropriate. This emphasizes the importance of paying close attention to the clinical features that raise the suspicion of malignant pseudoachalasia, even in the absence of diagnostic confirmation. It also indicates the necessity of timely upper endoscopic investigation with just as much importance on traversing the entire esophagus into the stomach as visualizing the anatomy itself. A collaborative effort is essential to optimal patient care, including timely input from pathology and radiology teams, as was the case with our patient. EUS is warranted if the diagnosis still remains suspicious. This case report is intended to educate clinicians on how to better diagnose and manage this often elusive condition.
